# Multi-functional metasurface as a transmissive/reflective FSS and an on-air frequency mixer

**DOI:** 10.1038/s41598-024-64306-y

**Published:** 2024-06-16

**Authors:** Anand Kumar, Saikiran Kongari, Yugesh Chandrakapure, Debdeep Sarkar

**Affiliations:** Department of Electrical Communication Engineering, Indian Institute of Science, Bengaluru, Bengaluru, KA 560012 India

**Keywords:** Electrical and electronic engineering, Techniques and instrumentation, Theory and computation

## Abstract

In this paper, a multi-functional metasurface is proposed, which can work as a narrowband transmissive/reflective frequency selective surface (FSS) and an on-air frequency mixer based on its switching response. The metasurface is made up of unit cells with square and circular metallic loops connected by PIN diodes controlled by a bias source. In contrast to typical wideband FSSs, the structure provides 0.55 GHz of narrow stopband (a fractional bandwidth of 22%) at 2 GHz in the OFF state bias. The bandstop response can be adjusted by varying the reverse bias voltage. The metasurface alternates between its functionalities when in forward bias by providing a passband at the operational frequency. The structure is compact and operates as a transmissive/reflective surface under two different bias conditions (ON and OFF). The design is angularly stable and polarization-insensitive for both TE and TM polarisation. A prototype of the designed structure is developed and the measured results correlate well with the simulated responses from the finite-difference time-domain (FDTD) method-based simulation of the circuit model. On-air frequency mixing for a wave propagating through the metasurface is demonstrated and the effects of different parameters affecting the mixing are parametrically studied through FDTD simulations and experiments.

## Introduction

With the fast growth of wireless technology, the issue of electromagnetic interference (EMI) among neighboring electronic gadgets and electrical machinery in sensitive surroundings has emerged^1,2^. An FSS may be widely used to conceal such components and is an excellent solution for many applications because of its low cost, small profile, and ease of production. Furthermore, FSS can provide band-pass or band-stop responses and these structures are frequently used in microwave and millimeter-wave regimes as spatial filters, antenna reflectors, radomes, absorbers, artificial magnetic conductors, etc^3,4^. Reducing interference and wireless security between neighboring wireless local area networks (WLAN) have been the focus of extensive research in recent years^5–8^.

**Figure 1:**
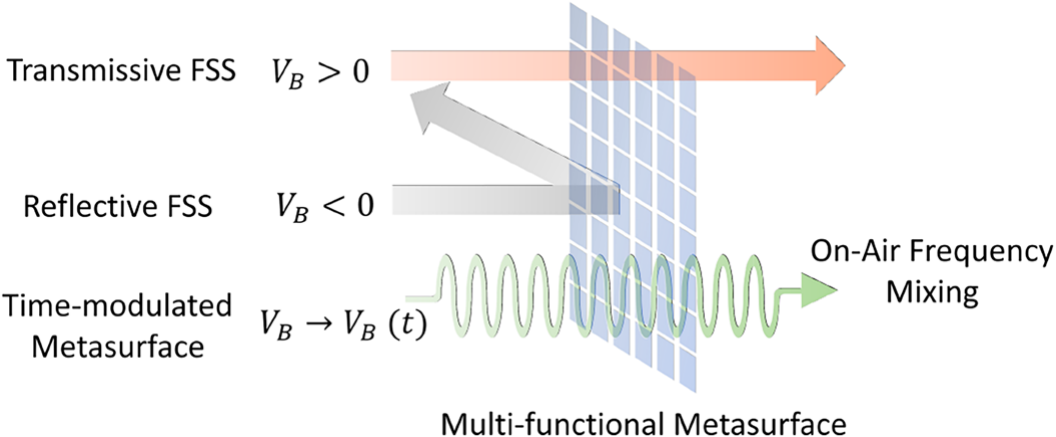
Conceptual illustration of multi-functionality of the proposed metasurface: Transmissive mode for ’ON’ state (bias voltage, $V_B>0$), Reflective mode for ’OFF’ state ($V_B<0$) and on-air frequency mixing for the transient state ($V_B \rightarrow V_B(t)$).

Active FSSs, which can function as reflective and transmissive surfaces using diodes^9–12^, have been recently developed to provide switchable frequency response. The frequency response can be varied with the control of the active devices incorporated into the periodic arrays. Active components, such as varactors and PIN diodes, enable high-speed and wideband tuning in a compact and low-cost package. The structure reported in^10^ can function in various modes but is polarization-sensitive. In^11^, an adjustable absorber is reported, but the structure has multiple layers. In^12^, the structure operates in different states, but the structure is time-invariant. Varactor-driven adjustable structures have recently become popular in offering shielding and polarization-insensitive behaviors^13,14^. However, in^13^, the structure is complicated since it calls for layer modifications based on the type of application, and the structure in^14^ is solely addressed for its shielding behavior. Therefore, it is necessary to develop a single structure without much complexity to achieve multiple functions such as various states (transmissive + reflective) as well as polarisation insensitivity.

Metasurfaces are two-dimensional metamaterials that are artificially engineered to offer unique electromagnetic properties (*“Meta”* = beyond)^15,16^. Time-engineered metamaterials/metasurfaces are metamaterials/metasurfaces whose constitutive parameters such as permittivity ($\epsilon$), permeability ($\mu$) or conductivity ($\sigma$) vary as a function of time^17–19^. The introduction of time-variation of media can lead to frequency translation and discrimination^20,21^, frequency mixing^19,22^ and non-reciprocity^23,24^. In^25^, an analysis of a free-space N-path-modulated metasurface is presented. In^26^, linear frequency conversion is demonstrated, and in^27^, a serrodyne frequency translation is proposed using a time-modulated metasurface. Time-modulated metasurfaces have been proposed for electromagnetic cloaking and defense applications, such as, in^28^, a time-modulated metasurface partially covering the targets is used to jam inverse synthetic aperture radar (ISAR) imaging and a target recognition based on Pseudorandom Noise sequence time-modulated metasurfaces in^29^ and phase-induced frequency conversion and Doppler effect with time-modulated metasurface in^30^. The works in^28,29,29,30^ are based on reflective structure, while the works in^9,26,27^ are transmissive surfaces.

In this paper, the authors have aimed to address some of the above-mentioned limitations by developing a single metasurface that can offer different functionalities based on its bias state. Figure 1 illustrates the multiple functions performed by the proposed metasurface under different bias conditions. Although the unit-cell reported in the present paper is inspired from prior works^14^, the multi-mode operation capability (i.e., spatial filtering and on-air frequency mixing modes of operation) is reported here for the first time, to the best of the authors’ knowledge. Another important advancement over existing literature^14^ is the explanation of the metasurface-based on-air frequency mixing with an in-depth analysis of the structure via simulations and measurements. Note that the existing commercial solvers have limited capability to model time-varying media properties. Therefore, the simulations for metasurface in frequency mixing mode and the investigation on the affecting parameters were performed using tailored finite-difference time-domain (FDTD) codes^31^. The proposed multi-functional metasurface was fabricated and successfully tested with a good correlation between simulated and measured results. The proposed work offers the following improvements over the earlier works:

- The proposed metasurface is simple in its design and operation and can function as a switchable transmission/reflection surface with a tunable frequency of operation and as an on-air frequency mixer. An incident wave propagating through the metasurface undergoes frequency mixing with the modulating frequency of the metasurface.
- The structure offers attenuation of more than 30 dB for a stop band of 0.55 GHz (22 % bandwidth), demonstrating its narrowband operation and tunability under different reverse bias voltages. Also, the design exhibits fourfold symmetry, providing tunable characteristics for all polarization angles.
- An FDTD model for the metasurface is developed from the circuit model to simulate time-varying capacitance and switchable functionality. Investigations of factors affecting frequency mixing were performed using FDTD simulations and measurements.

The paper is organized as follows. “Metasurface structure design” section describes the design of the proposed structure. “Equivalent circuit and the FDTD simulation model” section presents the development of the FDTD simulation model. In “Prototype fabrication and measurement results” section, the fabricated prototype of the metasurface and its measurement results are discussed. “Time-modulated metasurface for on-air frequency mixing” section discusses the on-air frequency mixing property and the parametric analysis of the affecting factors. Finally, the conclusion is presented in the last section.

## Metasurface structure design

The metasurface structure is intended to function as a transmissive/reflective surface at 2 GHz. Designing the structure’s OFF state model at a frequency lower than 2 GHz can increase the structure’s tuning range as the switches employed are the BAP64-03 Silicon PIN diodes, from NXP Semiconductors, which can operate up to 3 GHz^32^. These diodes possess variable capacitance and resistance under reverse and forward voltages, respectively. Whenever the reverse bias of the PIN diode rises, the intrinsic layer’s depletion region widens, and the diode’s capacitance decreases due to which the frequency of operation increases.

**Figure 2:**
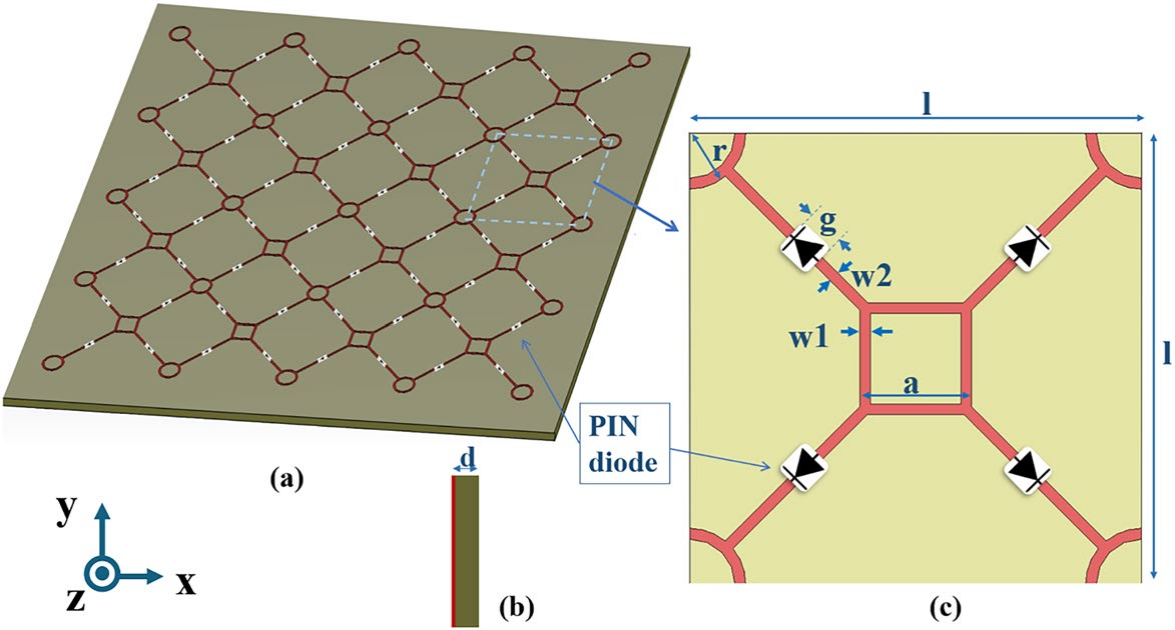
Schematic diagram of the proposed switchable metasurface prototype (**a**) Perspective view, (**b**) Side view and (**c**) Top view of the unit cell.

Figure 2 shows the array and unit cell shapes of the proposed multipurpose metasurface. A metallic square and circular loop design is printed in a regular pattern on a dielectric substrate for the top layer. Semiconductor switches that are diagonally arranged connect both square and circular loops. The dielectric substrate is FR4, with a relative permittivity ($\epsilon _r$) 4.4 and a loss tangent (tan$\delta$) of 0.02. The top metallic patch is made of copper, with a conductivity of $5.8 \times 10^7$ S/m and a thickness of 0.035 mm. The unit cell for the optimally designed structure has geometrical dimensions: l = 54 mm, a = 11 mm, r = 5.5 mm, w1 = w2 = 1.2 mm, g = 2 mm, and t = 1 mm. The incident field directions are also shown in Fig. 2. The operating frequency of the unit cell is controlled by the cell size (l = 54 mm, inter-spacing between the loops ) and the size of the loops (both a and r). The frequency ($\lambda _g$) of reflection or absorption by an FSS relies on the perimeter of patch loops in such a way that $\lambda _g$ is $2\pi r$ (or 4*a*), where *r* is the radius of the circular loop and *a* is the side of the square loop. The gap (g = 2 mm) is for placing the diode. In the absence of diodes, the air in this gap provides a low capacitance, which causes the structure to resonate at higher frequencies. The capacitance between the loops is varied by incorporating the PIN diodes in the gaps, which can offer lower capacitance ($\ll$ 1 pF). The required resonant frequency of 2 GHz is attained by optimizing the above parameters.

## Equivalent circuit and the FDTD simulation model

**Figure 3:**
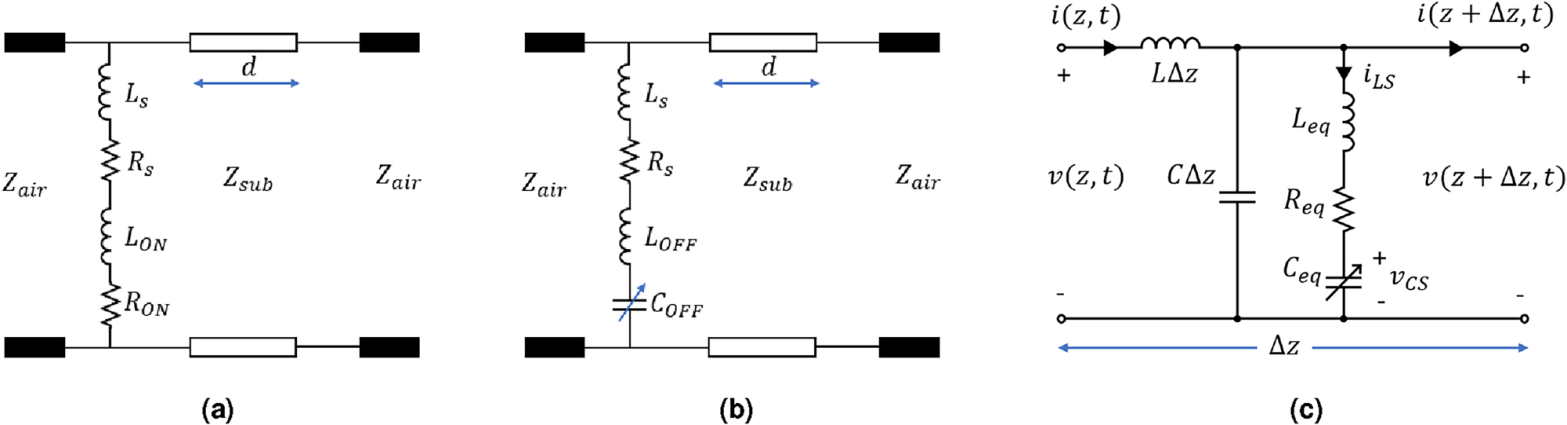
Equivalent circuit of the metasurface (suspended in air) in (**a**) ON state and (**b**) OFF state. (**c**) Generalized equivalent circuit for ON and OFF states.

The equivalent circuit of the proposed metasurface in ON and OFF states are shown in Fig. 3a,b, respectively. In the ON state (forward bias, $V_{B}>0~V$), the diode offers inductance ($L_{OFF}$) and resistance ($R_{ON}$), while in the OFF state (reverse bias, $V_{B}\le 0~V$), it offers inductance ($L_{OFF}$) and capacitance ($C_{OFF}$). $L_{s}$ and $L_{s}$ are the inductance and resistance of the metallic square/circular rings on the metasurface and $Z_{sub}$ is the impedance offered by the dielectric substrate. As the metasurface is placed in the air, either side of the circuit is terminated by $Z_{air}$ (=377 $\Omega$).

Figure 3c shows the modified unit cell of an incremental section of the transmission line developed to model the metasurface using the FDTD method based on the Telegrapher’s equation^33^. Applying KVL on the *RLC* branch yields,

1 $$\begin{aligned} i_{LS} R_{eq} + L_{eq} \frac{\partial i_{LS}}{\partial t}+v_{CS}-v(z+\Delta z,t)=0, \end{aligned}$$

where

2 $$\begin{aligned} v_{CS}(z+\Delta z,t)=\frac{1}{C_{eq}}\int _{t_{0}}^{t}i_{LS}(z+\Delta z,t)\partial t+v_{CS}(z+\Delta z,t_{0}). \end{aligned}$$

Applying KVL on the outer loop yields,

3 $$\begin{aligned} v(z+\Delta z,t)-v(z,t)+L\Delta z \frac{\partial i(z,t)}{\partial t}=0, \end{aligned}$$

and KCL on the top node yields,

4 $$\begin{aligned} i(z,t)-i(z+\Delta z,t)-c\Delta z \frac{\partial v(z+\Delta z,t)}{\partial t}-i_{LS}=0. \end{aligned}$$

Discretizing (1)-(4) using the central difference scheme yields the following FDTD update equations:

5 $$\begin{aligned} V_{CS}^{n}(k+1)&=V_{CS}^{n-1}(k+1)+\frac{\Delta t}{C_{eq}}I_{LS}^{n-0.5}(k+1), \end{aligned}$$

6 $$\begin{aligned} I_{LS}^{n+0.5}(k+1)&=\frac{\left( \frac {L_{eq}}{\Delta t}-\frac {R_{eq}}{2}\right) }{\left( \frac {L_{eq}}{\Delta t}+\frac {R_{eq}}{2}\right) }I_{LS}^{n-0.5}(k+1)+\frac{1}{\left( \frac {L_{eq}}{\Delta t}+\frac {R_{eq}}{2}\right) }\left[ V^{n}(k+1)-V_{CS}^{n}(k+1)\right] , \end{aligned}$$

7 $$\begin{aligned} I^{n+0.5}(k+ 0.5)&=I^{n-0.5}(k+0.5)-\frac{1}{Z_{c}} \frac{v_{p}\Delta t}{\Delta z}\left[ V^{n}(k+1)-V^{n}(k)\right] , \end{aligned}$$

8 $$\begin{aligned} V^{n+1}(k+1)&=V^{n}(k+1)-Z_{c}\frac{v_{p}\Delta t}{\Delta z}\left[ I^{n+0.5}(k+1.5)-I^{n+0.5}(k+0.5)-I_{LS}^{n+0.5}(k+1)\right] . \end{aligned}$$

### Update equations in the ON State

In the ON state, there is no $C_{eq}$, and hence $v_{CS}$ becomes 0. The resistance offered by the metallic rings is very low and hence $R_{eq}$ becomes $R_{ON}$ and $L_{eq}=L_{s}+L_{OFF}$. Therefore, the update equations in the ON state reduce to:

9 $$\begin{aligned} I_{LS}^{n+0.5}(k+1)&=\frac{\left( \frac {L_{eq}}{\Delta t}-\frac {R_{eq}}{2}\right) }{\left( \frac {L_{eq}}{\Delta t}+\frac {R_{eq}}{2}\right) }I_{LS}^{n-0.5}(k+1)+\frac{1}{\left( \frac {L_{eq}}{\Delta t}+\frac {R_{eq}}{2}\right) }\left[ V^{n}(k+1)\right] , \end{aligned}$$

10 $$\begin{aligned} I^{n+0.5}(k+ 0.5)&=I^{n-0.5}(k+0.5)-\frac{1}{Z_{c}} \frac{v_{p}\Delta t}{\Delta z}\left[ V^{n}(k+1)-V^{n}(k)\right] , \end{aligned}$$

11 $$\begin{aligned} V^{n+1}(k+1)&=V^{n}(k+1)-Z_{c}\frac{v_{p}\Delta t}{\Delta z}\left[ I^{n+0.5}(k+1.5)-I^{n+0.5}(k+0.5)-I_{LS}^{n+0.5}(k+1)\right] . \end{aligned}$$

### Update equations in the OFF state

In the OFF state $C_{eq}=C_{ON}$, $L_{eq}=L_{s}+L_{OFF}$, and as the resistance offered by the metallic rings is very low, for simplicity, $R_{eq}$ is taken to be 0. Therefore, the update equations in the OFF state reduce to:

12 $$\begin{aligned} V_{CS}^{n}(k+1)&=V_{CS}^{n-1}(k+1)+\frac{\Delta t}{C_{eq}}I_{LS}^{n-0.5}(k+1) \end{aligned}$$

13 $$\begin{aligned} I_{LS}^{n+0.5}(k+1)&=I_{LS}^{n-0.5}(k+1)+\frac{\Delta t}{L_{eq}}\left[ V^{n}(k+1)-V_{CS}^{n}(k+1)\right] , \end{aligned}$$

14 $$\begin{aligned} I^{n+0.5}(k+ 0.5)&=I^{n-0.5}(k+0.5)-\frac{1}{Z_{c}} \frac{v_{p}\Delta t}{\Delta z}\left[ V^{n}(k+1)-V^{n}(k)\right] , \end{aligned}$$

15 $$\begin{aligned} V^{n+1}(k+1)&=V^{n}(k+1)-Z_{c}\frac{v_{p}\Delta t}{\Delta z}\left[ I^{n+0.5}(k+1.5)-I^{n+0.5}(k+0.5)-I_{LS}^{n+0.5}(k+1)\right] . \end{aligned}$$

## Prototype fabrication and measurement results

**Figure 4:**
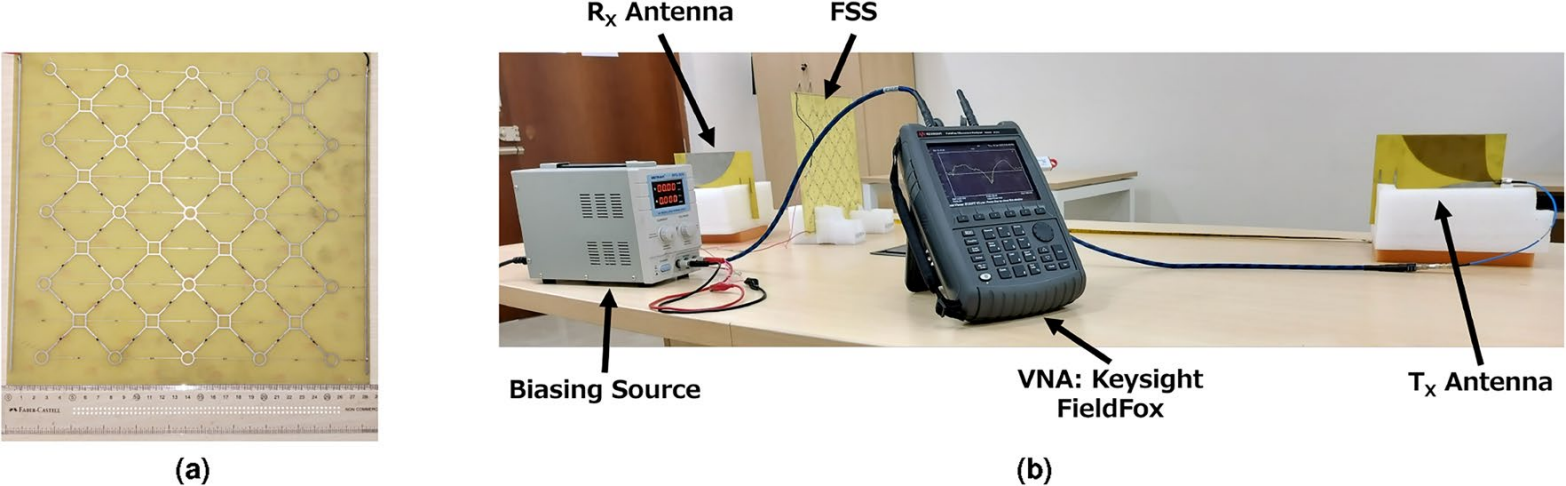
(**a**) Fabricated prototype of the Metasurface. (**b**) S-parameter Measurement setup for the metasurface.

The proposed metasurface, as stated in “Metasurface structure design” section, is fabricated and tested. DC bias lines are introduced in the structure to control the bias states of PIN diodes, as shown in Fig. 4a. The structure has physical dimensions of 290 mm $\times$ 275 mm, which consists of 4 $\times$ 4 arrays of unit cells. The measurement result shows that the structure operates at 2 GHz, slightly deviating from the simulation results, which can be accounted for by fabrication tolerance.

Circular loops at the outer edges of the unit cell are connected to the left-side bias line, whereas square loops in the unit cell’s center are connected to the right-side bias line. PIN diode’s anodes and cathodes are connected to the right and left bias lines by connecting them across the diagonal gap between the loops. Murata Electronics LQG18HH10NJ00D inductors with 10 nH values are used in consecutive central square loops to achieve isolation and protect the bias lines from EM interference. Bias lines allow direct current to flow continuously through PIN diodes. The resultant capacitance of the diodes about the DC bias voltage thus varies, leading to adjustable bandstop performance.

**Figure 5:**
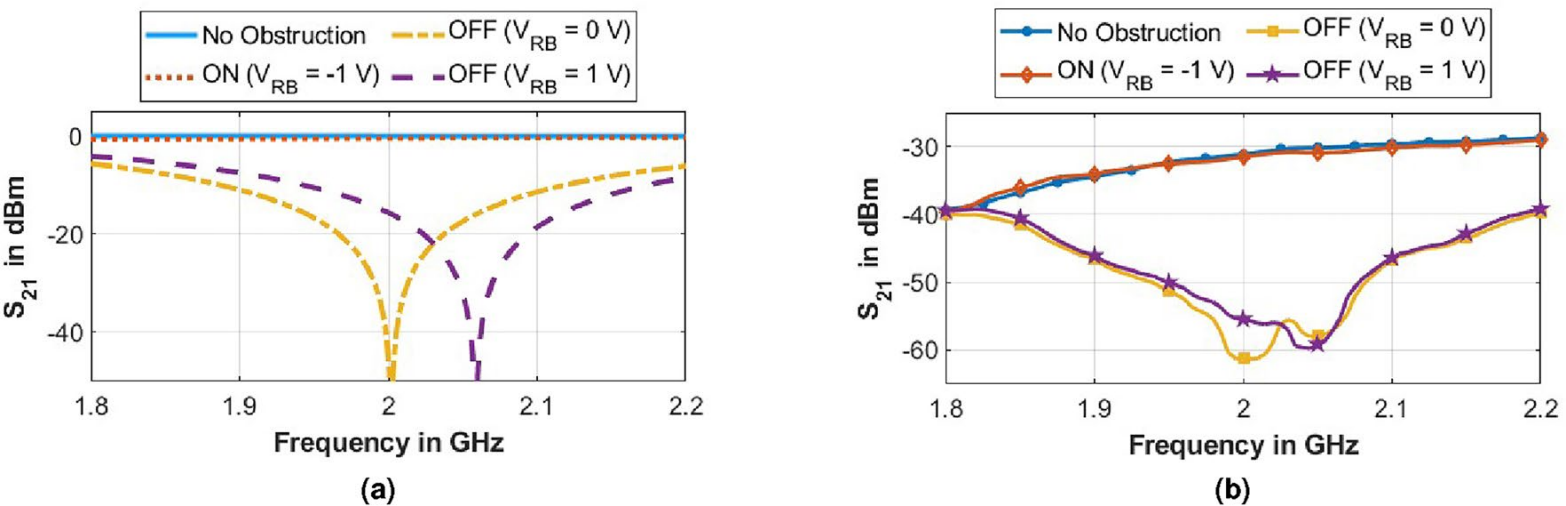
(**a**) Simulated (FDTD), and (**b**) measured $|S_{21}|$ for the proposed structure under different configurations.

The measurement setup is shown in Fig. 4b. It is organized by placing the testing structure between the two Vivaldi antennas facing each other. The metasurface is kept at the far field distance of the transmitting antenna. Both antennas are connected to the Vector Network Analyzer to estimate the structure’s reflection/transmission coefficient. At 2 GHz, when there is no obstruction, the power transmitted between the antennas is $-31$ dB from Fig. 5, which is close to the calculated value using the Friis Transmission formula. The transmission coefficients are measured between the antenna system in the subsequent steps under various configurations of the metasurface, shown in Fig. 5. The transmission coefficient of the metasurface is first determined in its OFF state. It is observed that the transmission coefficient is $-62$ dB at the desired frequency. It should be noted that the attenuation level is 30 dB, which is in agreement with the simulated results.

The shielding performance can be tuned to higher frequencies by varying the reverse bias voltage, as shown in Fig. 5. Shifting of shielding resonance to 2.06 GHz can be observed by applying a reverse bias voltage of 1 volt to the structure. Therefore, from the above observations, the structure is best suited for shielding applications in the open air at and above 2 GHz. Fabrication tolerances, parasitic capacitance, and inductance effects of PIN diodes account for the minor measurement discrepancies. The metasurface’s ON state performance is measured at 2 GHz using the same approach as the OFF state, except that forward bias is introduced into the structure. As seen in Fig. 5, the transmission coefficient is observed to be $-33$ dB. An attenuation of less than 2 dB is observed. This demonstrates the transmissive nature of the proposed structure. Therefore, the proposed structure performs a good switching response at 2 GHz in both ON and OFF states. Hence, the proposed metasurface works as an active transmissive/reflective FSS at 2 GHz and above.

**Figure 6:**
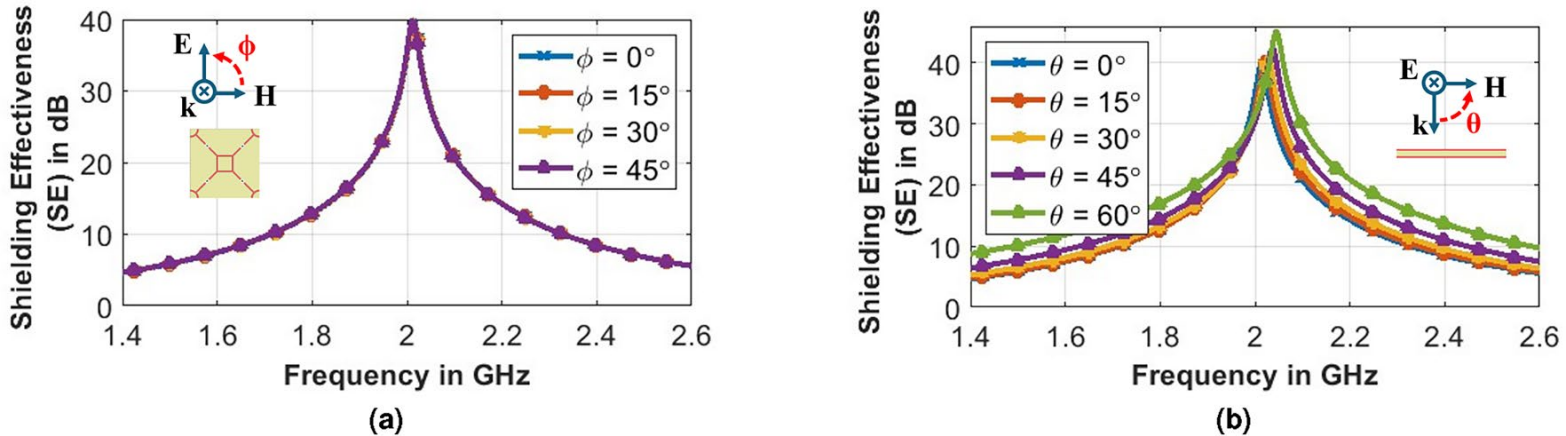
Shielding effectiveness of the proposed metasurface for different values of (**a**) polarization ($\phi$) and (**b**) oblique incidence angles ($\theta$).

The structure’s fourfold symmetrical design renders it polarization insensitive, ensuring consistent Shielding Effectiveness (SE) performance for all polarization angles ($SE_{dB} = -20 \times \log \left( \frac {E_t}{E_i}\right)$), as depicted in Fig. 6a. Furthermore, examination across various angles of oblique incidences, as illustrated in Fig. 6b, reveals a consistent stopband response up to a 60^∘^ angle of incidence.

## Time-modulated metasurface for on-air frequency mixing

**Figure 7:**
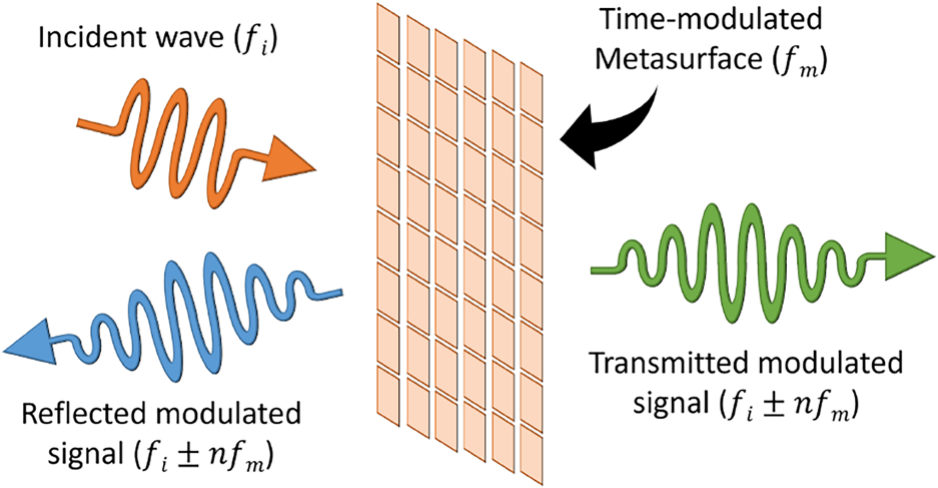
Illustration depicting on-air frequency mixing using time-modulated metasurface.

The proposed metasurface performs on-air frequency mixing when the metasurface is modulated with time. Figure 7 depicts frequency mixing where the incident wave of frequency $f_i$ is modulated by the metasurface operating at $f_m$. The reflected and transmitted waves contain new frequency components at $f_i \pm n f_m$ where $n = 1, 2, 3,$ and so on.

The variation of capacitance offered by the PIN diodes with time varies the surface impedance ($\eta _m$) of the metasurface with time, leading to a time-varying reflection coefficient ($\Gamma _m$) at the metasurface.

16 $$\begin{aligned} C_d \rightarrow C_d (t) \implies&\eta _m \rightarrow \eta _r (t) \end{aligned}$$

17 $$\begin{aligned} \implies&\Gamma _m \rightarrow \Gamma _m (t) = \bar{f_{\Gamma }}\{ \eta _r (t)\} \end{aligned}$$

18 $$\begin{aligned} ~&T_m \rightarrow T_m (t) = \bar{f_T}\{ \eta _r (t)\} \end{aligned}$$

Then the transmitted wave is given by $E_t (z,t) = \bar{f_{E_t}}\{ T_m (t), E_i (z,t) \}.$ Moving to the frequency domain, we get19Hence, (19) suggests that we will observe frequency mixing between the incident wave and the metasurface modulation frequency.

### Measurement setup and results

**Figure 8:**
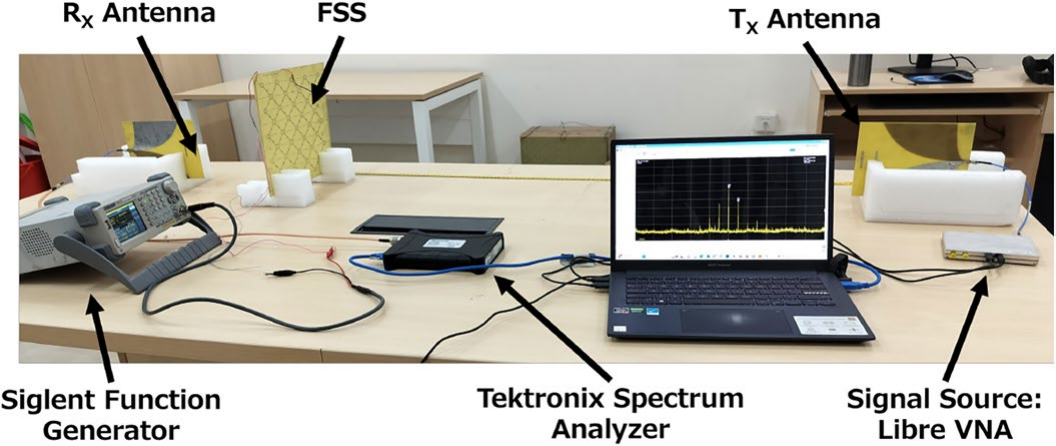
Measurement setup for testing of time-modulated metasurface in indoor scenario.

Figure 8 shows the measurement setup for demonstration of on-air mixing in an indoor scenario. A signal source from LibreVNA operating at $f_i$ is connected to antenna 1. The metasurface is biased using a Sigilent Function Generator to provide a modulating bias signal of bias voltage $V_B$ at frequency $f_m$. The receiver antenna 2 is connected to a Tektronix Real-time Spectrum Analyser. The metasurface is placed in the far field of the transmitting antenna for plane wave incidence while the receiving antenna is placed closer to the metasurface to reduce path loss in the measurement setup.

**Figure 9:**
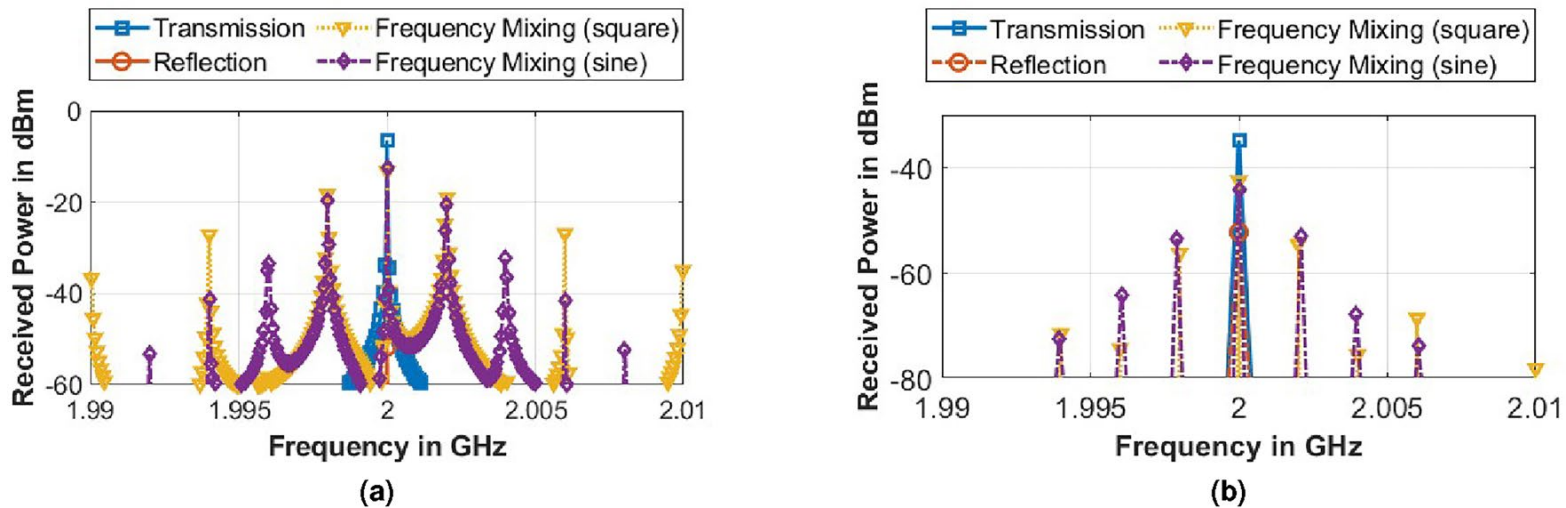
(**a**) Simulated (FDTD), and (**b**) measured spectrum for the received signal under different states of metasurface.

The frequency of the incident wave ($f_i$) on the metasurface is 2.4 GHz, and the spectrum of the received signal in simulation using the FDTD method is shown in Fig. 9a and the measured results in Fig. 9b when the modulation frequency of the metasurface $f_m$ was maintained at 2 MHz. The bias voltage $V_B$ of the metasurface is 2 V_pp_, and the power of the incident wave at the source is 0 dBm. While the unit-cell simulation of the FSS assumes an infinite periodic structure, the performance of a finite (4x4 size) metasurface tile is expected to resemble that of an infinite periodic structure under plane wavefront illumination, as in the shown experimental setup in Fig. 8. The metasurface tile and the Rx-antenna were positioned in the far-field region at a distance of 1.2 m, well beyond the 30 cm far-field distance at 2 GHz. Moreover, the directive radiation of the Tx and Rx Vivaldi antennas helps minimize edge diffraction effects from the finite-sized tile. Consequently, we observe reasonable consistency between simulation and measurement results, encompassing both band-stop response and on-air mixing due to time modulation.

### Parametric analysis

The on-air frequency mixing using time-modulated metasurface demonstrated in Fig. 9 is further analyzed using square wave and sinusoidal modulation of the metasurface via parametric analysis of the following parameters:

#### Variation of incident frequency ($f_i$)

**Figure 10:**
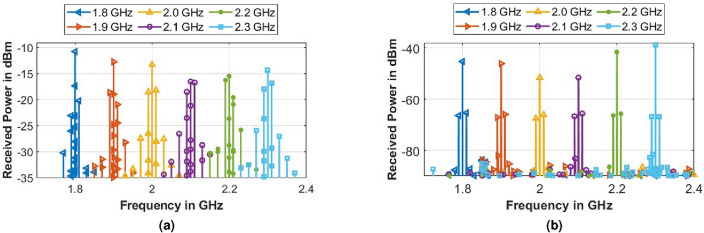
(**a**) Simulated (FDTD), and (**b**) measured spectrum for the received signal for variation of incident frequency ($f_i$).

The frequency of the incident wave ($f_i$) on the metasurface was varied, keeping $f_m$ = 10 MHz, $V_B$ = 2 V_pp_ and transmitted power at the incident frequency $|P(f_i)|$ = 0 dBm and the spectrum of the received signal is shown in Fig. 10. It is observed that the newly generated frequencies correspond to $f_i \pm n f_m$, and the power at the incident frequency $f_i$ is minimum near the resonance of the metasurface while the power at the new modulated frequencies does not vary with the change of $f_i$.

**Figure 11:**
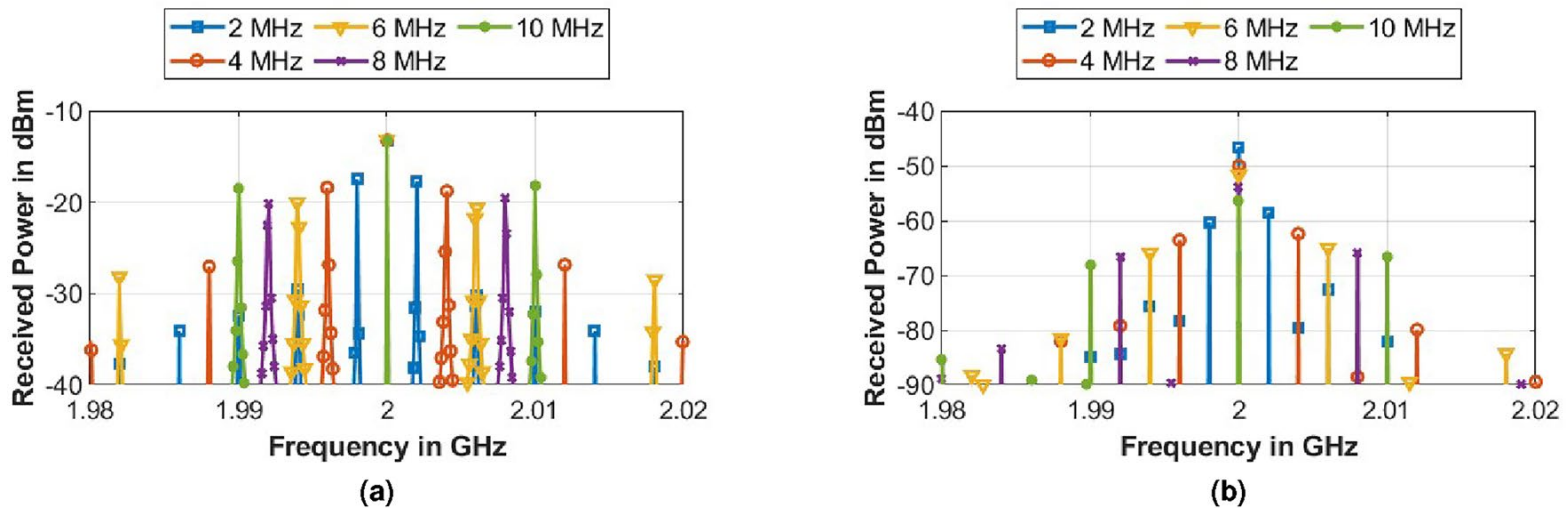
(**a**) Simulated (FDTD), and (**b**) measured spectrum for the received signal for variation of metasurface modulation frequency ($f_m$).

**Figure 12:**
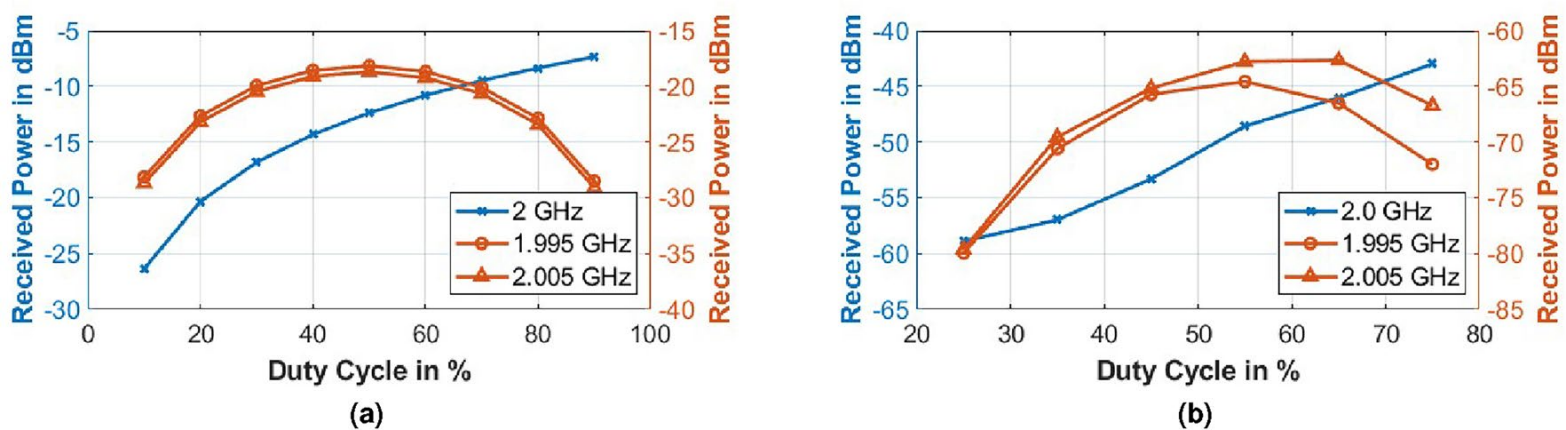
(**a**) Simulated (FDTD), and (**b**) measured power for the received signal v/s variation of duty cycle (*DC*) at incident frequency ($f_i$) and achieved modulated frequencies ($f_i \pm f_m$).

**Figure 13:**
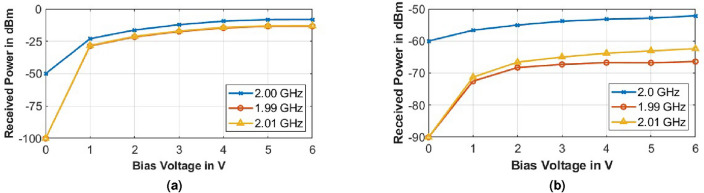
(**a**) Simulated (FDTD), and (**b**) measured received signal power v/s metasurface bias voltage ($V_B$) at incident frequency ($f_i$) and achieved modulated frequencies ($f_i \pm f_m$).

**Figure 14:**
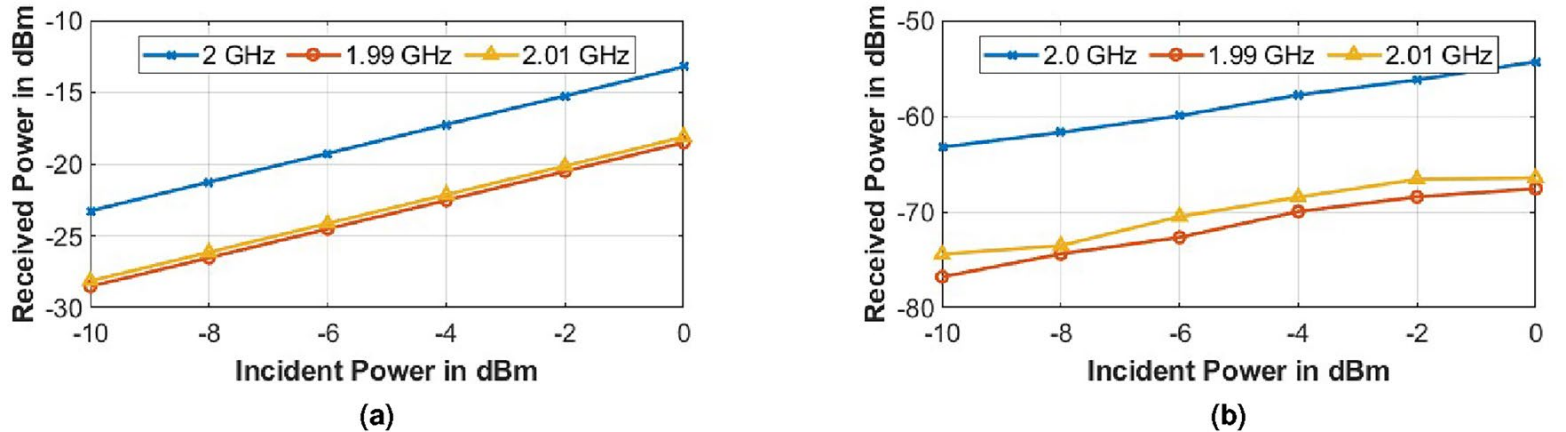
(**a**) Simulated (FDTD), and (**b**) measured received signal power v/s Incident power on the metasurface bias voltage ($P_in$) at the incident frequency ($f_i$) and achieved modulated frequencies ($f_i \pm f_m$).

#### Variation of metasurface modulation frequency ($f_m$)

Similarly, the metasurface modulation frequency $f_m$ was varied keeping $f_i$ as 2.4 GHz, $|P(f_i)|$ = 0 dBm and $V_B$ = 2 V_pp_. It is observed that the newly generated frequencies correspond to $f_i \pm n f_m$, where *n* = 1, 2, 3...and the power in the new frequencies and their harmonics decrease as we move away from $f_i$. It was also observed that in the case of modulation of metasurface via square wave, the power received at the incident frequency $f_i$ decreases with an increase in modulation frequency $f_m$ while for a sinusoidal modulation, it showed negligible variation as shown in Fig. 11.

#### Variation of duty cycle (*DC*)

For modulation of the metasurface via a square wave of frequency 5 MHz, the duty cycle *DC* of the wave was varied from 25 to 75%, thereby varying the transmission to blocking duration ratio. From Fig. 12, it is observed that the power of the received wave at incident frequency 2 GHz increases with an increase in the DC as the ON-state favors transmission, but the power in the modulated peaks initially increases with the DC, achieving a maximum around 50–60% and then decreases with further increase in DC.

#### Variation of metasurface bias voltage ($V_B$)

The amplitude of the bias voltage $V_B$ of the metasurface at frequency $f_m$ was varied. The variation of bias voltage affects the range of impedance change ($\eta _{m2} / \eta _{m1}$) offered by the metasurface, where $\eta _{m1}$ is the minimum surface impedance while $\eta _{m2}$ is the maximum. From Fig. 13, it is observed that with an increase in $V_B$ the received power at the newly generated frequencies $f_i \pm f_m$ increases rapidly initially and then gradually saturates, while power at $f_i$ remains almost constant.

#### Variation of power of incident frequency ($|P(f_i)|$)

Figure 14 shows the plot for variation of received power at $f_i$ = 2.4 GHz, $f_i-f_m$ = 2.31 GHz and $f_i+f_m$ = 2.41 GHz with the power of incident wave at $f_i$ at the source. The power at the source was varied from $-20$ to 0 dBm while keeping $V_B$ = 2 V_pp_. It is observed that power at $f_i$ and $f_i \pm f_m$ vary linearly with the increase in incident power.

The FDTD-based framework aims to predict the on-air mixing capabilities for metasurface illuminated by TEM waves. However, FDTD-based simulation considers TEM waves in a guided wave scenario with negligible path loss and linear time-varying behavior of the capacitance, whereas the measurements are done using propagating TEM waves with free-space path loss (FSPL) and non-linear response of the PIN diodes. Therefore, while the mixed frequency components are correctly predicted, the observed power levels show inconsistency. The FSPL value for the proposed experimental setup depicted in Fig. 8 is $\approx$ 28 dB at 2 GHz. This accounts for the diminished power received in the measured results compared to the simulated results.

**Table 1:** Comparison of proposed metasurface with recent literature.

Ref.	Operating Freq. (in GHz)	Type	Mode	Description
^3^	6.81–18.97 22.28–55.78	Passive	Transmissive	Wide-bandstop FSS resonating at X-band, Ku-band and millimeter-wave applications
^5^	4.8–11.1	Passive	Reflective	Ultra-broadband thin FSS based radar absorber
^6^	2.5 and 5.45	Passive	Reflective	Ultra-miniaturised Polarisation Selective Surface (PSS) for dual-band Wi-Fi and WLAN shielding applications
^9^	9.2	Active	Transmissive	Dynamic control of asymmetric EM wave transmission
^10^	8.61 and 11.33	Re-configurable	Reflective + Transmissive	Multi-functional re-configurable FSS
^13^	10	Re-configurable	Reflective + Transmissive	Tuneable and broadband absorber using a switchable transmissive/reflective FSS
^14^	2.5	Re-configurable	Reflective + Transmissive	Broadband Polarization-Insensitive Tunable FSS for Wideband Shielding
^25^	10	Active	Reflective	Analysis of a free-space N-path-modulated metasurface
^26^	2.3, 3.3, and 5	Active	Transmissive	Spurious-free and linear frequency-conversion
^27^	10	Active	Transmissive	Serrodyne frequency translator
^28^	10	Active	Reflective	Jamming of inverse synthetic aperture radar (ISAR) imaging with time-modulated metasurface partially covered on targets
^29^	2.4	Active	Reflective	Target recognition based on Pseudo-random Noise sequence time-modulated metasurfaces
^30^	1.5	Active	Reflective	Phase-induced frequency conversion and Doppler effect
This work	2	Active + Re-configurable	Reflective + Transmissive	Narrowband re-configurable transmissive/reflective FSS and on-air frequency mixing

A comparison of the developed multi-functional metasurface with recent literature is presented in Table 1. The studies referenced in^3,9,25–30^ present active/passive metasurfaces where^3,9,26,27^ operate solely in transmissive mode whereas those in^25,28–30^ function exclusively in reflective mode. The studies in^10,13,14^ present re-configurable FSSs operating in both transmissive and reflective modes. In contrast, our work introduces an active re-configurable multi-mode metasurface capable of operating in both reflective and transmissive modes. This metasurface serves as a narrowband transmissive/reflective FSS and functions as an on-air frequency mixer.

## Conclusion

This work proposes an active multifunctional metasurface with a tuneable narrowband reflective/transmissive response and demonstrates on-air frequency mixing. The proposed metasurface prototypes are fabricated and tested. The simulated and measured results show that the metasurface has a switchable performance between nearly transparent and total reflection. Furthermore, the reported reflective/transmissive surface demonstrated narrowband absorption with a $-10$ dB bandwidth of up to 0.55 GHz in the vicinity of 2 GHz. The structure suggested can provide a wide tuning range for similar bandstop responsiveness as a function of reverse bias voltage. Furthermore, the structure has the advantages of reduced geometry, polarisation insensitivity, and angular stability. Also, a significant degree of agreement between simulation and experimental results was obtained. The concept has been expanded to demonstrate on-air frequency mixing. Additionally, the effect of parameters such as incident frequency, metasurface modulation frequency, bias voltage, and incident power on the frequency mixing have been analyzed via parametric study. To the best of the authors’ knowledge, no singular structure demonstrating multi-mode operation as an electromagnetically transparent, opaque, and on-air frequency mixer using a transmissive-type metasurface has been reported in the literature. Furthermore, FDTD-based simulation for prediction of on-air mixing by the metasurface is also reported for the first time.

## Data Availability

All data generated or analysed during this study are included in this published article.
